# Rapid-onset dystonia-parkinsonism associated with the I758S mutation of the *ATP1A3* gene: a neuropathologic and neuroanatomical study of four siblings

**DOI:** 10.1007/s00401-014-1279-x

**Published:** 2014-05-07

**Authors:** Adrian L. Oblak, Matthew C. Hagen, Kathleen J. Sweadner, Ihtsham Haq, Christopher T. Whitlow, Joseph A. Maldjian, Francine Epperson, Jared F. Cook, Mark Stacy, Jill R. Murrell, Laurie J. Ozelius, Allison Brashear, Bernardino Ghetti

**Affiliations:** 1 Department of Pathology and Laboratory Medicine, Indiana University School of Medicine, Indianapolis, IN USA; 2 Department of Pathology and Laboratory Medicine, University of Cincinnati College of Medicine, Cincinnati, OH USA; 3 Department of Neurosurgery, Massachusetts General Hospital, Harvard Medical School, Boston, MA USA; 4 Department of Neurology, Wake Forest School of Medicine, Wake Forest Baptist Health, Winston-Salem, NC USA; 5 Department of Radiology (Neuroradiology), Wake Forest Baptist Health, Winston-Salem, NC USA; 6 Department of Neurology, Duke University School of Medicine, Duke Health, Durham, NC USA; 7 Department of Genetics and Genomic Sciences and Neurology, Icahn School of Medicine at Mount Sinai, New York, NY USA

**Keywords:** *DYT12*, RDP, Rapid-onset dystonia-parkinsonism, Neuropathology

## Abstract

Rapid-onset dystonia-parkinsonism (RDP) is a movement disorder associated with mutations in the *ATP1A3* gene. Signs and symptoms of RDP commonly occur in adolescence or early adulthood and can be triggered by physical or psychological stress. Mutations in *ATP1A3* are also associated with alternating hemiplegia of childhood (AHC). The neuropathologic substrate of these conditions is unknown. The central nervous system of four siblings, three affected by RDP and one asymptomatic, all carrying the I758S mutation in the *ATP1A3* gene, was analyzed. This neuropathologic study is the first carried out in *ATP1A3* mutation carriers, whether affected by RDP or AHC. Symptoms began in the third decade of life for two subjects and in the fifth for another. The present investigation aimed at identifying, in mutation carriers, anatomical areas potentially affected and contributing to RDP pathogenesis. Comorbid conditions, including cerebrovascular disease and Alzheimer disease, were evident in all subjects. We evaluated areas that may be relevant to RDP separately from those affected by the comorbid conditions. Anatomical areas identified as potential targets of I758S mutation were globus pallidus, subthalamic nucleus, red nucleus, inferior olivary nucleus, cerebellar Purkinje and granule cell layers, and dentate nucleus. Involvement of subcortical white matter tracts was also evident. Furthermore, in the spinal cord, a loss of dorsal column fibers was noted. This study has identified RDP-associated pathology in neuronal populations, which are part of complex motor and sensory loops. Their involvement would cause an interruption of cerebral and cerebellar connections which are essential for maintenance of motor control.

**Electronic supplementary material:**

The online version of this article (doi:10.1007/s00401-014-1279-x) contains supplementary material, which is available to authorized users.

## Introduction

Rapid-onset dystonia-parkinsonism (RDP) is a rare disease associated with mutations in the *ATP1A3* gene [3, 6, 11, 19, 30, 35, 39], located on chromosome 19. RDP is inherited with an autosomal dominant pattern and incomplete penetrance. Recently, *ATP1A3* mutations have been shown to be also associated with alternating hemiplegia of childhood (AHC), which is characterized by early paroxysmal events, developmental delay and later dystonia, ataxia, choreoathetosis and seizures [26]. The Na^+^/K^+^-ATPase alpha subunit 3 (ATP1A3) is one of the essential components that maintain the proper sodium and potassium gradients across cell membranes [11].

Epidemiological data related to RDP and AHC are limited and there is not a recently published report summarizing the known data. The number of individuals affected by RDP is estimated to be 75 worldwide (A.B., personal communication) while AHC is estimated to affect one person in one million [25]. RDP and AHC are associated to *ATP1A3* gene mutations. The number of mutations is 14 for RDP [3, 6, 11, 19, 30, 35, 39] and 37 for AHC [12, 14, 18, 30–32]. Both RDP and AHC may be associated with de novo mutations.

The clinical phenotype of RDP is typically characterized by abrupt onset of dysarthria, dysphagia, limb dystonia with bradykinesia, and postural instability [13]. In a study of 29 patients with mutations in the *ATP1A3* gene, 76 % had onset of motor symptoms before the age of 25 years [7]. Other symptoms, including anxiety, cognitive impairment [10] and psychosis, have recently been reported [2, 7]. The reported age range for onset of RDP is 9 months [6] to 59 years [2]. Most patients present with speech (38 %) and arm (5 %) dystonia; however, leg involvement alone has also been reported [6, 36]. Onset of symptoms in RDP has often been related to physical and psychological stresses. The former includes running, fever, childbirth, and alcohol abuse. Recognized symptom onset occurs shortly following one of these specific events. Some at risk individuals have been exposed to similar triggers, but never developed symptoms of the disease. Most patients with RDP have persistent symptoms over time; however, some individuals have reported an additional episode of abrupt worsening of symptoms. Fluctuating motor symptoms have been observed in the two infants with RDP, previously reported [6].

Structural imaging has provided some insight into central nervous system areas that might be potentially affected in RDP. In fact, Whitlow and colleagues [37] studied three subjects carrying mutations in *ATP1A3* gene and found significant gray matter volume and fractional anisotropy (FA) decreases in the parahippocampal gyrus and dentate nucleus, unilaterally. Fractional anisotropy was also decreased in the superior cerebellar peduncles, thalami, basal ganglia (caudate nucleus, putamen, and globus pallidus), internal and external capsules, and corpus callosum [37].

The present study details the neuropathologic and the molecular genetic findings in four siblings, three affected and one asymptomatic, all carrying the I758S mutation in the *ATP1A3* gene. These subjects are members of a large family originally reported by Dobyns et al. [13]. The mutation in members of this family was originally reported by de Carvalho-Aguiar [11]. The clinical phenotype associated with the I758S mutation is similar to that seen in individuals affected by RDP but carrying different *ATP1A3* gene mutations.

In consideration of the fact that in the individuals described in this study, RDP manifests itself early in life and has a life-long duration, identifying the anatomical abnormality and related histopathology may be challenging due to the fact that the neuropathologic studies are being carried out many years since the beginning of symptomatology. Therefore, we made an effort to distinguish the alterations of gray and white matter structures that we hypothesize to be underlying RDP, from vascular and neurodegenerative pathology, associated with aging and Alzheimer disease. This study is the first describing anatomical alterations in *ATP1A3* mutation carriers.

## Methods

### Clinical assessments

The pedigree, to which the four individuals that are the object of the present study belong, was included in the first paper describing RDP [13]. In that study, one family member, later found to carry the I758S mutation in the *ATP1A3* gene, was described. That individual and the additional three siblings, all mutation carriers, were subsequently examined over the following 25 years. Each subject signed an IRB-approved Wake Forest School of Medicine Consent. Furthermore, all individuals signed the appropriate autopsy consent forms several years prior to their death. The clinical assessment carried out by A.B. included a standardized patient history questionnaire covering RDP antecedent symptoms, triggers, motor and non-motor symptoms, and a standardized videotaped neurologic examination including the Unified Parkinson’s Disease Rating Scale [24] and the Burke–Fahn–Marsden [8] dystonia rating scale. The videos and scales were blindly reviewed by an expert in dystonia (M.S.).

### Psychiatric and cognitive testing

Formal psychiatric and cognitive evaluations were performed on individuals capable of participating. These evaluations have been described in prior reports [7, 10].

### Neuropathologic methods

Neuropathologic studies were carried out on all four subjects. Table 1 includes information about the four subjects’ (case 1, 2, 3, 4) gender, age of onset, mutation status, age at death, post-mortem interval, brain weight, Alzheimer disease changes and *Apolipoprotein E* (*APOE*) alleles. In addition, neuropathologic analysis was carried out on the brain of 16 controls cases (Supplementary Table 1). These controls were selected on the basis of age and comorbid conditions.

**Table 1:** Demographic information for each subject

Case number	Sex	Age of onset	Age at death	Affected (Y/N)	Mutation carrier	PMI (h)	Brain weight (g)	Alzheimer Disease changes (A, B, C)	APOE
1	F	28	81	Yes	Yes	9.0	1,144	3, 2 ,1	33
2	F	25 and 42	82	Yes	Yes	12.0	1,128*	3, 2, 2	33
3	F	n/a	87	No	Yes	9.0	1,076	3, 2, 3	33
4	M	45	85	Yes	Yes	5.0	1,226	3, 2, 2	23

* The brain weight of case 2 is an estimate

Autopsy from all four subjects and controls was limited to the brain and spinal cord; cases 1 and 3 were harvested at Indiana University and cases 2 and 4 were harvested at other institutions. At the time of autopsy, the brains of case 1, 3, 4 and the controls were hemisected along the mid-sagittal plane. The left hemibrain and spinal cord were fixed in formalin and the right hemibrain was sliced, frozen, and stored at −70 °C. For case 2, the anterior part of the frontal lobe (bilaterally), the cerebellum, and the brainstem were frozen and stored at −70 °C; the remaining portions, including the spinal cord, were fixed in formalin.

**Fig. 1:**
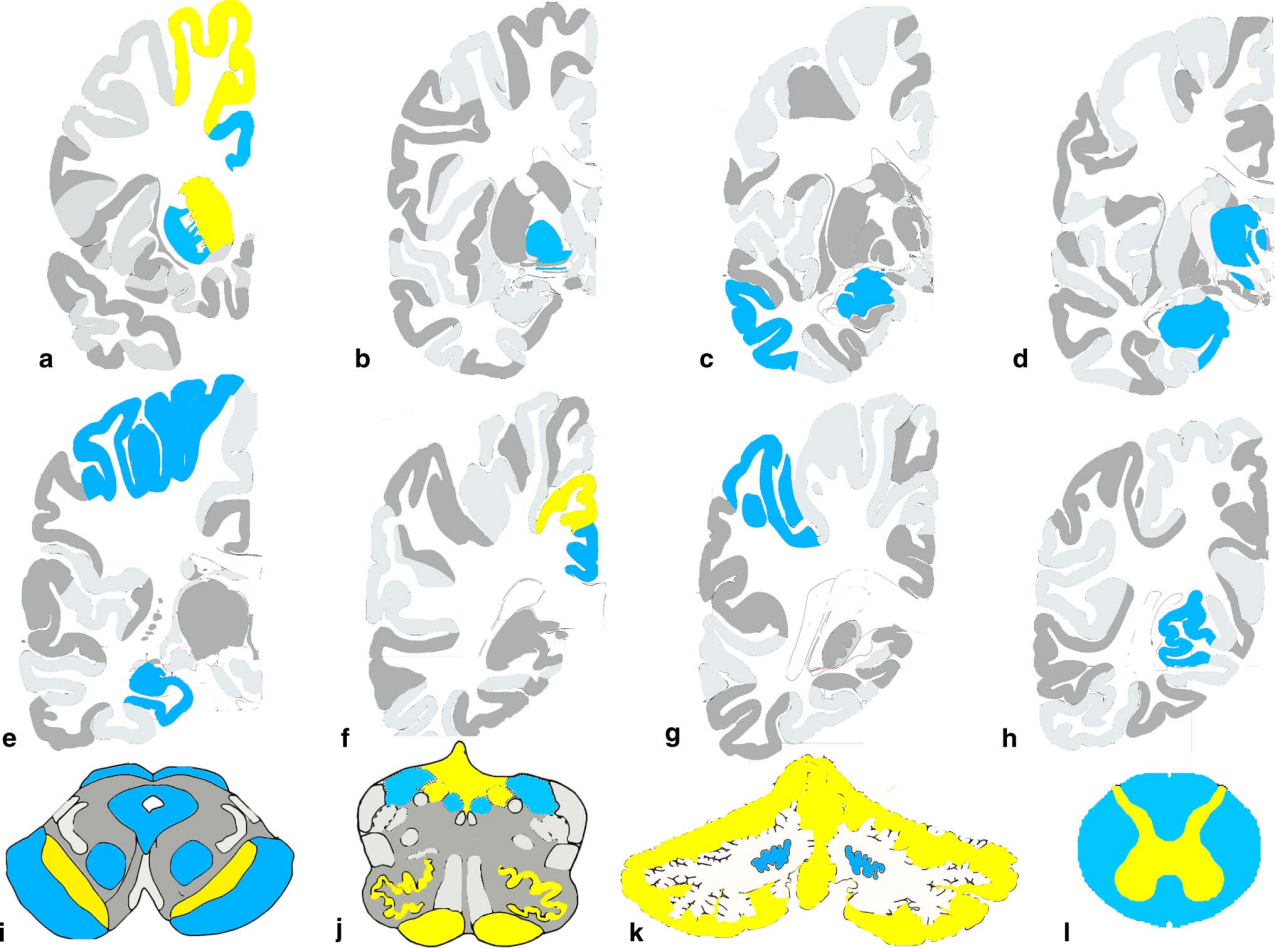
**a**–**h** The brain areas from which samples for neuropathologic studies were obtained. Brain areas highlighted in (**a**–**h**) (*blue* or *yellow*) represent the following regions: anterior cingulate gyrus, superior frontal gyrus, middle frontal gyrus, caudate nucleus, putamen, globus pallidus, substantia innominata, amygdala, superior temporal gyrus, middle temporal gyrus, insula, thalamus, subthalamic nucleus, superior parietal lobule, hippocampus, posterior cingulate cortex, precuneus, inferior parietal lobule, calcarine cortex. Sections were also taken at the level of the midbrain (**i**), medulla (**j**), dentate nucleus of the cerebellum (**k**), and spinal cord (**l**). Coronal sections were obtained at the levels **a** to **f**. Images adapted from The Atlas of the Human Brain 3rd Edition [21]

Following fixation in a 10 % formalin solution, brain tissue samples listed in Table 2 were dehydrated in graded alcohols, cleared in xylene, and embedded in paraffin (see Fig. 1 for summary of tissue blocks obtained). Eight-micrometer-thick sections from the brain areas listed in Table 2 were stained with the histological and immunohistochemical methods described below. Hematoxylin and eosin (H&E) and luxol fast blue with hematoxylin & eosin (LFB-H&E) were used to survey gray and white matter for neuronal losses, gliosis, inflammatory changes, vascular pathology, and other possible pathologic lesions. Periodic acid–Schiff (PAS) was used to rule out the presence of PAS-positive cellular or extracellular deposits. Heidenhain-Woelcke was used to determine whether a loss of myelin or myelinated fibers was detectable. Bodian silver was selected to analyze neurons and their processes for the presence of abnormal fibrillar structures. Thioflavin S method was used to visualize amyloid deposits and neurofibrillary tangles. Neurodegenerative pathology was analyzed using antibodies raised against amyloid beta (Aβ) (21F12), tau (AT8), glial fibrillary acidic protein (GFAP), α-synuclein [28], phosphorylated TDP-43 and ubiquitin. In the cerebellum, synaptophysin was used to detect possible synaptic losses while calbindin [9] was mainly used to investigate the status of the Purkinje cell somata and that of the dendrites in the molecular layer and axons throughout their course.

**Table 2:**
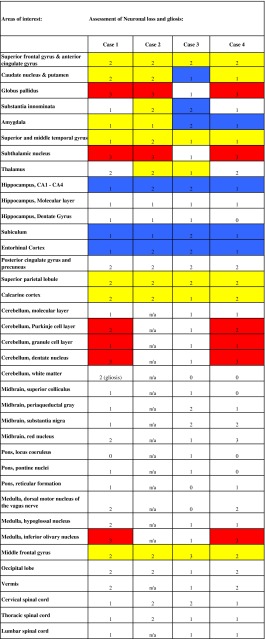
Neuropathologic assessment for each subject by brain area

Degree of neuron loss and gliosis in brain regions: none = 0, mild = 1, moderate = 2, severe = 3

Areas of neuronal loss and gliosis, and no associated AD-related pathology (neuritic plaques, diffuse plaques, neurofibrillary tangles, neuropil threads, tau-ir neurons)

Neuritic and/or diffuse plaques present

Neuritic and/or diffuse plaques and neurofibrillary tangles present

The signal from polyclonal antibodies was visualized using avidin–biotin, with goat anti-rabbit immunoglobulin as the secondary antibody, followed by horseradish peroxidase-conjugated streptavidin and the chromogens diaminobenzidine or tetramethylbenzidine. The signal from monoclonal antibodies was detected using avidin–biotin, with goat anti-mouse immunoglobulin as the secondary antibody, followed by alkaline phosphatase-conjugated streptavidin and the chromogen diaminobenzidine or tetramethylbenzidine. Immunohistochemical sections were counterstained with hematoxylin.

Immunofluorescence in the cerebellum of the symptomatic mutation carriers, non-symptomatic mutation carrier, and controls was carried out. Slides were deparaffinized through graded xylenes, and antigen retrieval was performed with hot 10 mM Tris base and 1 mM disodium EDTA pH 9.0 at 60 °C for 10 min. Slides were incubated with antibody as above, except that incubation with primary antibody (rabbit antibody from Santa Cruz Biotechnology, sc-16052, C-16, against Na,K-ATPase alpha3) was for 72 h. After washing, incubation with Alexa dye-conjugated fluorescent antibodies (Life Technologies) was for 2 h, followed by additional washes. Fluorescence was evaluated with a Zeiss LSM 5 Pascal laser confocal microscope with the Zeiss RGB vario laser module of argon laser (458/488/514 nm) and two helium–neon lasers (543 and 633 nm).

In the four cases, we also obtained coronal sections of cerebral hemispheres as well as parasagittal sections of the cerebellum in three of the cases, to analyze in greater detail the gray and white matter pathology. Sections were stained using LFB-H&E as well as immunolabeled using antibodies against GFAP and Thioflavin S. Coronal sections of the cerebral hemisphere were not obtained in the 16 controls.

Three investigators trained in anatomy and neuropathology (AO, MH, BG) carried out a survey of anatomical structures, independently, and reached a consensus. A semi-quantitative analysis was carried out using a Leica DMLB microscope (Leica Wetzlar, Germany), from frontal cortex, cingulate cortex, temporal cortex, parietal cortex, insular cortex, occipital cortex, amygdala, hippocampus, subiculum, parahippocampus, caudate nucleus, putamen, globus pallidus, thalamus, cerebellar hemisphere, dentate nucleus, substantia nigra, locus coeruleus, basis pontis, inferior olivary nucleus, and spinal cord. The severity of neuronal loss, gliosis, amyloid plaques, neurofibrillary tangles, and tau-immunoreactive neurons was evaluated using a subjective scale: none = 0, mild = 1 (+), moderate = 2 (++) and severe = 3 (+++). In view of the presence of Alzheimer changes, we classified each case according to criteria of CERAD [22], Braak staging [5], and NIA-AA [17, 23].

The Atlas of the Human Brain 3rd Edition [21] was adapted to map the loss of neurons, gliosis, and type of deposited protein (Aβ plaques, and neurofibrillary tangles) changes in the anatomical regions of interest.

### Molecular genetic analysis

To detect the presence of the I758S mutation in the brain of the four siblings, DNA was extracted from samples of frozen cerebellum. Qiagen DNeasy Blood and Tissue Kit was used to isolate the DNA according to manufacturer’s instructions. PCR amplification of exon 17 of the *ATP1A3* gene was performed using primers and conditions specified previously [11], and the mutation was confirmed by Sanger sequencing. DNA was extracted from frozen tissue using standard procedures. *APOE* genotypes were determined by restriction enzyme digestion of amplified products [16].

## Results

### Clinical observation

For the cases presented, we refer to the original pedigree from Dobyns et al. [13]. Structural imaging data could not be obtained due to technical difficulties related to the patients’ advanced stage of disease and comorbid conditions.

#### Case 1 (case II.4)

Within an hour after giving birth to her fourth child, at age 28 the subject exhibited an oculogyric crisis and gaping mouth. The oculogyric crisis, a rarely observed symptom in RDP, resolved after a few hours but she remained unable to talk or swallow. She had severe upper limb dystonia and was unable to write. She was sent to a psychiatric hospital for several months where she was treated with electroconvulsive therapy (ECT). It is reported that she gradually improved to the point where she could feed herself but the food had to be liquefied. She was able to walk with assistance with a short shuffling gait. She remained stable until age 50 when she experienced severe psychological stress. This triggered a second episode, resulting in worsening over 6 months followed by a gradual deterioration over 10 years, including becoming wheelchair-bound. Examination at age 80 found her to be alert and cooperative but completely anarthric, requiring a touch screen communication tool. She was fed with a feeding tube due to dysphagia causing aspiration. Both arms exhibited severe dystonia, the left being more affected than the right with marked bradykinesia. She was unable to stand without assistance due to marked postural instability. She had cogwheel rigidity, bradykinesia in the right arm and leg, but no tremor. Formal psychiatric and cognitive evaluations were not performed due to motor and vocal limitations (Supplementary Table 2).

#### Case 2 (case II.3)

This subject is the identical twin of case 1. This patient had ECT for depression some time prior to onset of symptoms. At age 25, following the birth of a child, her left arm became dystonic over the course of a month and she developed a limp, though was able to walk independently. Her symptoms remained stable until age 42 when she experienced severe psychological stress and showed a worsening of her gait, as well as abnormalities in her voice and her balance over 1–2 weeks. She could still walk unassisted but with a very broad-based gait and marked postural instability. Her swallowing worsened, limiting her diet to puréed foods. At age 81, she remained unable to talk. Her left arm was rigid, and the right arm demonstrated cogwheel rigidity, but no tremor. There was marked bradykinesia of finger movements in both hands, and the left hand had no useful grasp. She could no longer walk, but could rise and transfer chairs with difficulty. Formal psychiatric and cognitive evaluations were not performed due to motor and vocal limitations (Supplementary Table 2).

#### Case 3 (case II.1)

This subject was asymptomatic but had three affected descendants. She had no problems with movement, but had leg cramps that were treated with quinine. She had congestive heart failure and had been previously treated with digitalis (a specific inhibitor of Na^+^/K^+^-ATPase that does cross the blood–brain barrier to some degree). She did not remember any adverse effects with this medication. Her last examination, at age 84, demonstrated slowness in movement, the absence of tremor, and normal gait. She reported that she did not have tremors but complained about feeling overall weak due to failing health.

No illness was found on psychiatric assessment. The patient did report that she was treated for depression following the death of her son (Supplementary Table 2). Caution is warranted in interpreting cognitive testing (Supplementary Table 3) as poor visual acuity was noted upon examination. Despite her reported memory problems, verbal list learning was intact. Visual memory for abstract designs was reduced. Psychomotor processing speed was impaired. She displayed mild impairment on tasks of linguistic and semantic verbal fluency.

#### Case 4 (case 11.2)

At age 45, symptoms began gradually during a self-reported emotionally stressful time. Symptoms started in the right hand over 2 weeks and progressed to the right leg over 1 year. He reported heavy smoking and alcohol use. At age 53–54, he reported a worsening of symptoms following psychological stress. He was also treated for depression and took digitalis for heart disease. At age 83, a neurological examination revealed a soft, low volume speech, cogwheel rigidity, subtle non-focal weakness in both arms and legs, and a resting tremor in the right hand. Definite dystonic movements in hands and feet were present.

Upon psychiatric assessment, he received diagnoses of psychotic disorder not otherwise specified (NOS), panic disorder without agoraphobia, and alcohol abuse. Symptoms of psychosis included paranoid delusions, thought insertion, thought broadcasting, and thought withdrawal (Supplementary Table 2).

Overall cognitive skills were estimated to be below average (Supplementary Table 3). Memory was generally within normal limits. Delayed recognition of a word list was notable for numerous false-positive errors, suggesting poor discrimination. Executive functioning was reduced. Psychomotor processing speed was within normal limits, though performance on the motorically demanding symbol digit modalities test (SDMT: written and oral) was impaired.

### Neuropathology

A summary of case demographics is provided in Table 1. A detailed case-by-case description follows.

### Macroscopic observation

*Case 1* The brain weighed 1,144 g. No focal lesions were observed over the lateral or medial surface of the brain. Atherosclerosis was observed in the basilar and the middle cerebral arteries. The cerebrum was mildly and diffusely atrophic. The lateral ventricles were slightly enlarged. No changes were detected in the subcortical white matter. The corpus callosum measured 9 cm in the antero-posterior dimension; its thickness was 12 mm at the genu, 5, 9 and 4 mm at the anterior, middle and posterior portion of the body, and 12 mm at the splenium. The head of the caudate nucleus, the putamen, and the globus pallidus were reduced in bulk. The thalamus was unremarkable. The subthalamic nucleus was thin and gray in appearance and measured 1 mm in its widest dimension. The amygdala and hippocampus were unremarkable. The rostral portion of the cerebellar vermis was mildly atrophic. In the midbrain, the substantia nigra appeared reduced in width and its pigmentation was uneven. In the pons, the locus coeruleus was well pigmented. No macroscopic abnormalities were observed in the spinal cord.

*Case 2* The brain weight, estimated by adding the weight of fixed and frozen portions of the entire cerebrum, cerebellum and brainstem, was 1,128 g. No focal lesions were observed over the lateral or medial surface of the brain. The status of the major vessels of the base of the brain could not be ascertained. Mild atrophy of the frontal and temporal lobes was noted. The lateral ventricles were unremarkable. The white matter of the centrum semiovale and of the temporal lobe was reduced in bulk. Measurements of the corpus callosum were considered to be unreliable due to modifications of the specimen’s shape in transportation. The caudate nucleus, putamen and thalamus were unremarkable. The globus pallidus was moderately atrophic. The amygdala and hippocampus were mildly atrophic. A macroscopic evaluation could not be carried out on the cerebellum and most of the brainstem. The anterior portion of the substantia nigra was well pigmented. No macroscopic abnormalities were observed in the spinal cord.

*Case 3* The brain weighed 1,076 g. No focal lesions were observed over the lateral or medial surface of the brain. Severe atherosclerosis was observed in the vertebral, basilar, internal carotid and middle cerebral arteries. There was mild atrophy of the frontal, temporal, and parietal lobes. The lateral ventricles were unremarkable. The centrum semiovale was reduced in bulk. The corpus callosum thickness was 10 mm at the genu, 7 and 4 mm at the body anteriorly and posteriorly, and 10 mm at the splenium. The caudate nucleus, putamen, and globus pallidus were unremarkable. There was a mild reduction in bulk of the thalamus. The subthalamic nucleus was unremarkable. The cerebellar vermis appeared mildly to moderately atrophic. The substantia nigra and locus coeruleus were well pigmented. No macroscopic abnormalities were observed in the spinal cord.

*Case 4* The brain weighed 1,226 g. No focal lesions were observed over the lateral or medial surface of the brain. Atherosclerosis was observed in the posterior and middle cerebral arteries. The cerebrum showed mild diffuse atrophy. The lateral ventricles were unremarkable. There was a reduction in bulk of the white matter of the frontal lobe. The corpus callosum measured 10 cm in its antero-posterior length; the thickness of the corpus callosum was 12 mm at the genu, 7, 10 and 4 mm at the anterior, middle and posterior portion of the body, and 13 mm at the splenium. The head of the caudate nucleus, the putamen, the globus pallidus, and the subthalamic nucleus were mildly atrophic. The cerebellar vermis and dentate nucleus were atrophic. The substantia nigra was mildly depigmented. Mild, diffuse atrophy of the pons was observed. The locus coeruleus was well pigmented. No macroscopic abnormalities were observed in the spinal cord.

### Microscopic changes

A systematic evaluation led to the observation of neuropathologic changes in several areas; however, they represented alterations classifiable within different disease categories. The following fundamental pathologic processes were detected: cerebrovascular disease, neurodegenerative disease of the Alzheimer type, as well as neuronal atrophy and loss in specific subcortical nuclei. In Table 2, we have summarized and separated neurodegenerative disease of the Alzheimer type from potentially RDP-related neuronal loss and gliosis.

#### Cerebrovascular pathology

All cases showed non-occlusive, mild to moderate atherosclerosis. Atherosclerosis of the basilar, posterior cerebral and middle cerebral arteries was found in case 1. In case 2, arteriolosclerosis was observed in the arterioles of the neocortex, caudate nucleus, putamen, and hippocampus. Atherosclerosis was observed in the vertebral, basilar, internal carotid and middle cerebral arteries of case 3; arteriolosclerosis was observed in the arterioles of the neocortex. In case 4, atherosclerosis was found in the posterior and middle cerebral arteries; arteriolosclerosis was observed in the arterioles of the neocortex, caudate nucleus, putamen, and hippocampus.

#### Neurodegenerative changes of Alzheimer type

**Fig. 2:**
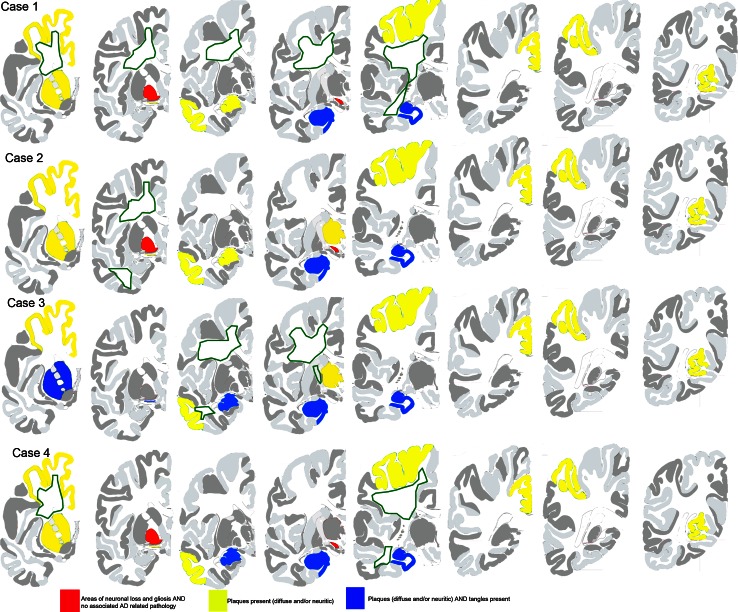
Summary of neuropathologic findings from the cerebral hemisphere of each case (adapted from [21]). Brains areas that are colored (*yellow*, *blue*, or *red*) demonstrate regions with neuronal loss and gliosis. *Red* represents areas that do not have neurodegenerative changes of the Alzheimer type. *Yellow* represents areas where diffuse and/or neuritic plaques were found. *Blue* represents areas where diffuse and/or neuritic plaques and neurofibrillary tangles were present. *Green dashed lines* represent areas of the subcortical white matter where axon and myelin loss was found

**Fig. 3:**
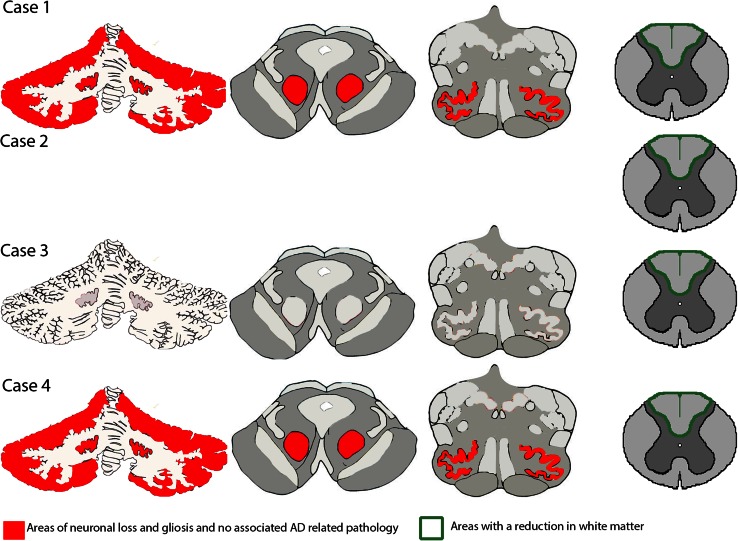
Summary of neuropathologic findings from the cerebellum, brainstem, and spinal cord of each case (adapted from [21]). *Red* represents areas that have neuronal loss and gliosis but do not have neurodegenerative changes of the Alzheimer type. The *green* outline represents loss of white matter

Bodian silver stain and Thioflavin S method as well as immunohistochemistry using antibodies to Aβ (21F12) and tau (AT8) revealed Alzheimer disease pathology in all cases. The degree of neuropathologic changes varied for severity and distribution (see Figs. 2, 3).

Case 1 was classified as having the following Alzheimer Disease Neuropathologic Change: A3, B2, C1, intermediate likelihood. Mild to moderate amyloid angiopathy was observed in vessels of the parenchyma of the caudate nucleus, putamen, superior parietal lobule, and calcarine cortex.

Case 2 was classified as having the following Alzheimer Disease Neuropathologic Change: A3, B2, C2, intermediate likelihood. Mild to moderate amyloid angiopathy was observed in vessels of the parenchyma of the caudate nucleus, putamen, thalamus, subiculum, entorhinal cortex, and calcarine cortex.

Case 3 was classified as having the following Alzheimer Disease Neuropathologic Change: A3, B2, C3, intermediate likelihood. Moderate to severe amyloid angiopathy was observed in vessels of the parenchyma of the temporal cortex, superior parietal lobule, and calcarine cortex.

Case 4 was classified as having the following Alzheimer Disease Neuropathologic Change: A3, B2, C2, intermediate likelihood. Severe amyloid angiopathy was observed in vessels of the parenchyma of the amygdala, temporal cortex, hippocampus, superior parietal lobule, calcarine cortex, and cerebellum.

Immunohistochemistry for α-synuclein and TDP-43 was negative for all cases.

#### Neuronal loss and gliosis in areas affected by neurodegenerative changes of the Alzheimer type

Loss of neurons and gliosis coexisted with AD pathology in several cortical and subcortical regions to varying degrees in each case (Table 2). In cases 1, 2, and 4, areas affected by neuronal loss and gliosis were the superior frontal gyrus, middle frontal gyrus, cingulate gyrus, superior temporal gyrus, middle temporal gyrus, calcarine cortex, superior parietal lobule, subiculum, parahippocampus, entorhinal cortex, thalamus, caudate nucleus, putamen, substantia innominata, amygdala, and hippocampus. In case 3, neuronal loss and gliosis were noted in the same areas but to a lesser degree.

#### Neuronal loss and gliosis in areas free from neurodegenerative changes of the Alzheimer type

**Fig. 4:**
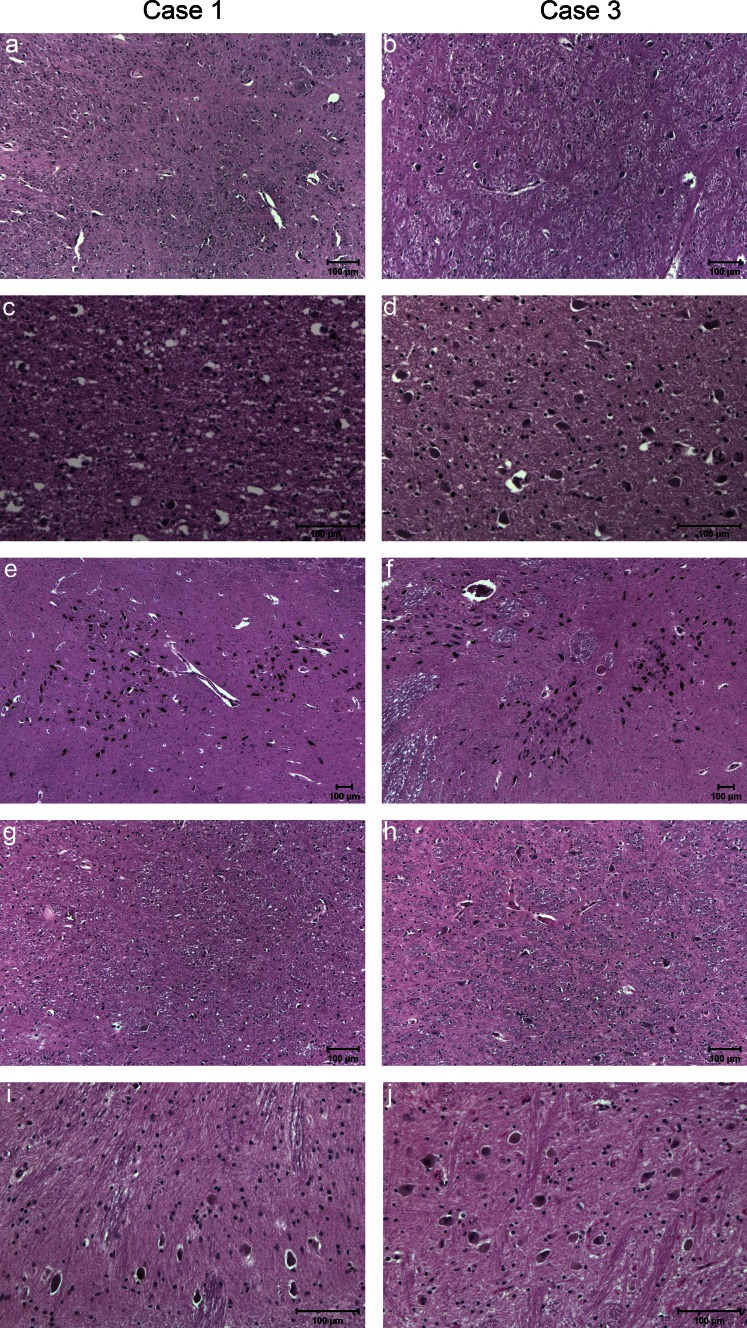
Hematoxylin & eosin (H&E) staining from cases 1 (**a**, **c**, **e**, **g**, **i**) and 3 (**b**, **d**, **f**, **h**, **j**). Note the extensive cell loss in the globus pallidus and subthalamic nucleus of case 1 (**a**, **c**) compared to the same areas in case 3 (**b**, **d**). **e**, **f** The preservation of cells in the substantia nigra. There are severe neuron loss and gliosis in the red nucleus in case 1 (**g**) compared to case 3 (**h**). **i**, **j** The severity of neuron loss and the reduced size of neurons in the inferior olivary nucleus of the affected (**i**) compared to the unaffected (**j**)

In the symptomatic siblings, loss of neurons and gliosis were observed in the globus pallidus and subthalamic nucleus. In the brainstem (available only from cases 1 and 4), neuron loss and gliosis were observed in the red nucleus, superior colliculus, periaqueductal gray matter, pontine nuclei (case 1), reticular formation, dorsal motor nucleus of the vagus nerve, hypoglossal nucleus, and inferior olivary nucleus (Table 2; Fig. 4a–j). Similarly, loss of neurons and gliosis were observed in the same nuclei of the asymptomatic sibling, but to a lesser degree (Fig. 4; Table 2).

In the portion of the cerebral peduncle corresponding to the cortico-spinal tract, a loss of myelinated fibers was observed in cases 1 and 4 (not shown, but similar deficits are shown in Fig. 6 below).

**Fig. 5:**
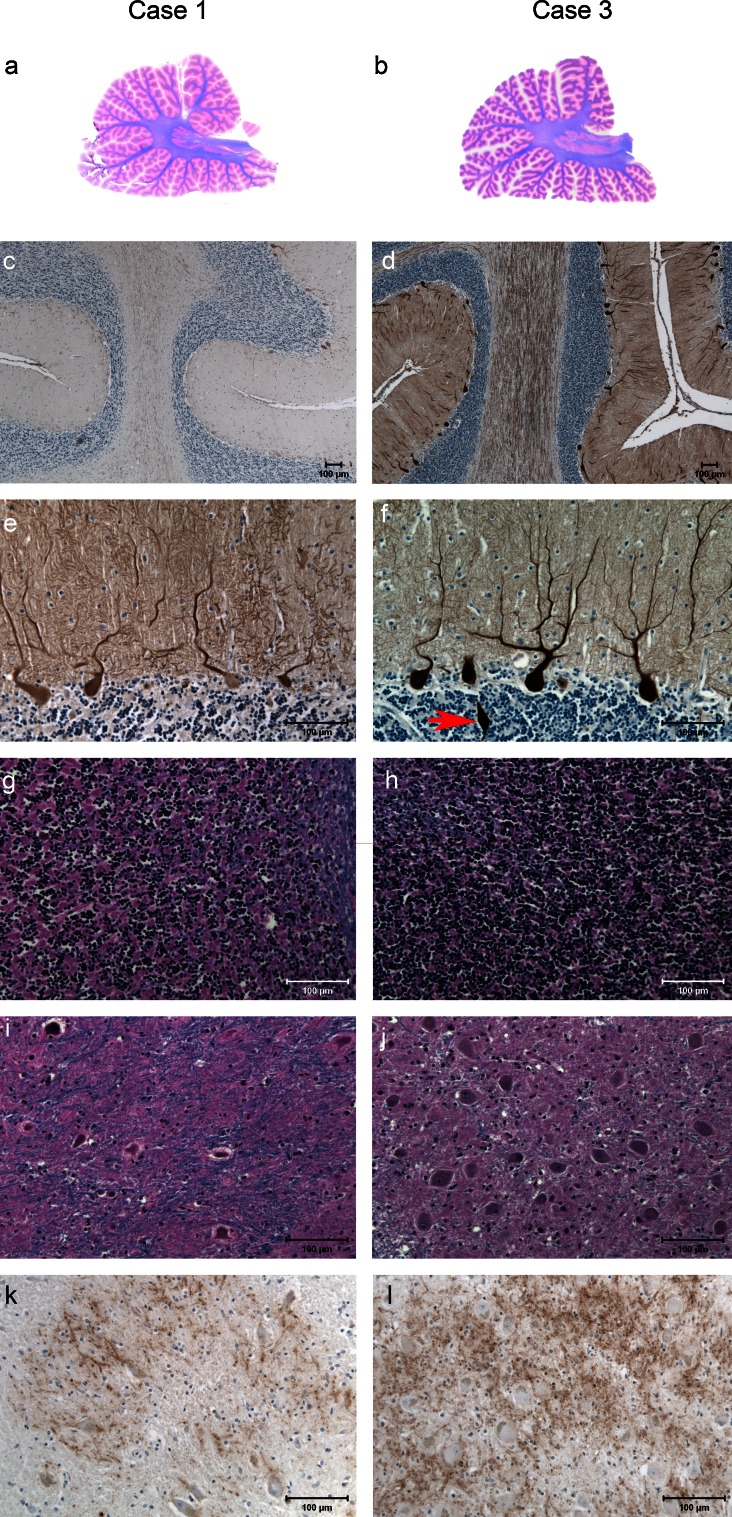
Images show a comparison of cerebellar morphology in case 1 (**a**, **c**, **e**, **g**, **i**, **k**) with that of case 3 (**b**, **d**, **f**, **h**, **j**, **l**). **a**, **b** Parasagittal sections of the cerebellar hemisphere. Note the mild atrophy of the cerebellar cortex and pallor of the cerebellar white matter. **c**–**f** Calbindin-immunolabeled preparations. Note the loss of Purkinje cell bodies and axons in **c** and loss of dendritic arborization in **e** as compared to **d** and **f**. Note an axonal swelling in **f** (*red arrow*). Note the reduction in granule cells in **g** as compared to **h**. Note the depletion of nerve cell bodies in the dentate nucleus in **i** as compared to **j**. There is a reduction in synapthophysin immunohistochemical labeling in the dentate nucleus in **k** compared to **l**

Histologic sections from the cerebellum were available from symptomatic cases 1 and 4 and asymptomatic case 3. Purkinje cells, granule cells, and neurons in the dentate nucleus appeared reduced in number compared to the asymptomatic sibling (Fig. 5e–j). Gliosis was present in the cerebellar cortex and intrafoliar white matter. Axonal and myelin loss was present. Swellings of axons of the Purkinje cells (torpedoes) were observed in the granule cell layer and dentate nucleus of symptomatic and asymptomatic siblings. Calbindin immunohistochemistry revealed a reduction in dendritic arborization of Purkinje cells as well as axonal swellings (see arrow Fig. 5f) and an apparent reduction in fibers of the white matter of the cerebellar folia (Fig. 5c, d). There was a reduction in synaptophysin immunochemical labeling in the dentate nucleus of the affected siblings compared to the controls (Fig. 5k–l).

A portion of the spinal cord was available from each case. There was a loss of fibers of the dorsal columns.

#### Subcortical white matter

**Fig. 6:**
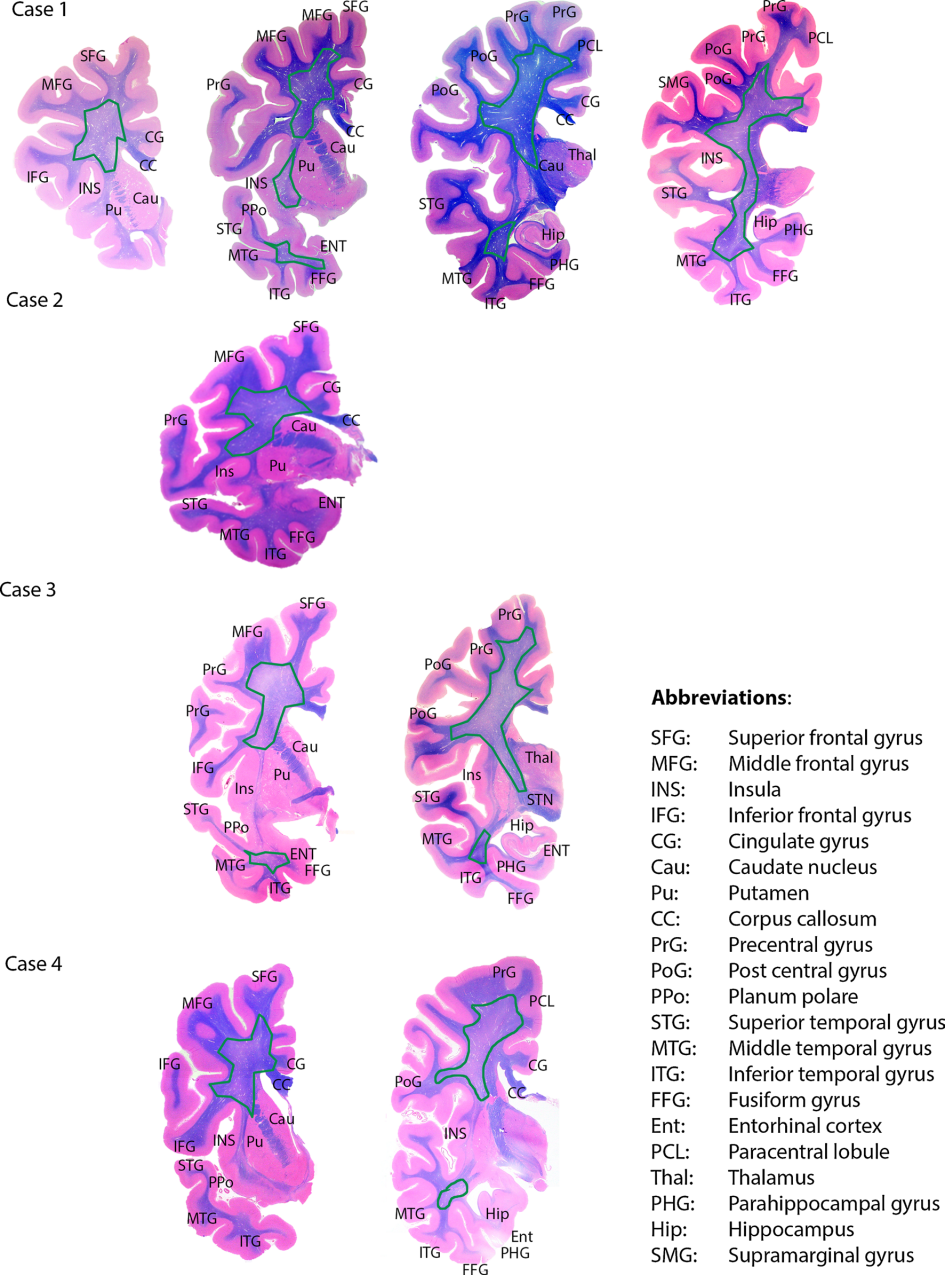
Hematoxylin & eosin/luxol fast blue staining of hemispheric coronal sections. All four cases showed pallor of the white matter in the frontal and temporal regions. The white matter tracts affected include the internal capsule, corona radiata, superior frontal occipital fasciculus, superior longitudinal fasciculus, inferior frontal occipital fasciculus, and inferior longitudinal fasciculus. These areas are outlined in *green*

In all four cases, axon and myelin loss was observed in the subcortical white matter of the middle frontal gyrus, inferior frontal gyrus, pre-central gyrus, post-central gyrus, middle temporal gyrus, and inferior temporal gyrus (Fig. 6). The white matter tracts affected are located in the areas corresponding to the following white matter tracts: anterior limb of the internal capsule, anterior region of the corona radiata, superior region of the internal capsule, superior frontal occipital fasciculus, superior longitudinal fasciculus, inferior frontal occipital fasciculus, and inferior longitudinal fasciculus [15, 33]. In addition, the corpus callosum of the four siblings was reduced in thickness (Fig. 6).

**Fig. 7:**
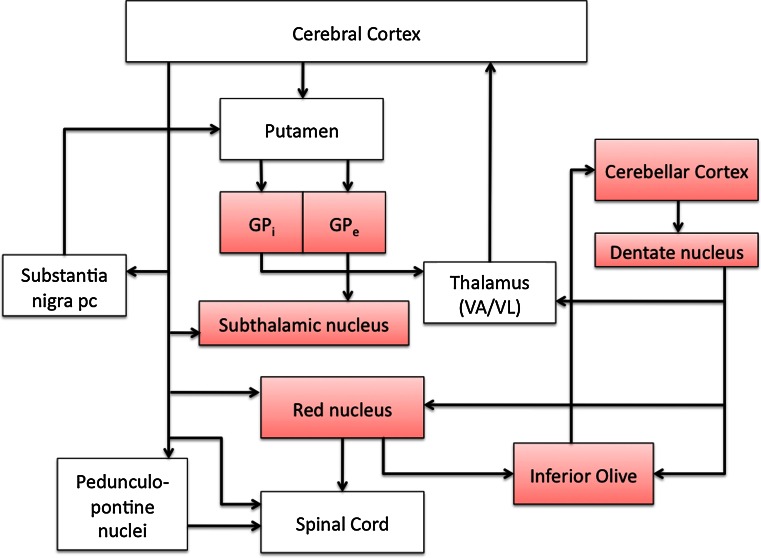
Circuit diagram depicting basal ganglia, thalamic, cortical, cerebellar, and brainstem connections. *Red* shading highlights the regions of the circuit where neuron loss and gliosis were found in the symptomatic siblings. The pathways affected are the pallido-thalamo-cortical loop, subthalamic-pallido-thalamo-cortical loop, dentato-thalamo-cortical loop, dentato-rubral loop, and dentato-olivary loop

As a summary, we show a complex system of connections highlighting nuclear groups most likely involved in RDP (Fig. 7).

### Controls

Each of the four subjects carrying the I758S mutation was analyzed against a set of 16 control cases. The control cases were divided in four groups. Cases of each group had the same degree of Alzheimer disease and cerebrovascular pathology as each of the four subjects. Alzheimer changes involved the same cortical areas as in the I758S mutation carriers. The distribution of AD pathology was comparable in RDP and control subjects (see Table 2 and Supplementary Table 1). Unlike what was observed in the symptomatic RDP patients, the globus pallidus, subthalamic nucleus, red nucleus, inferior olivary nucleus, Purkinje cell layer, granule cell layer, and dentate nucleus in individuals with Alzheimer disease and vascular disease only, as well as in the I758S non-symptomatic mutation carrier, showed a lesser degree of gliosis and cell loss compatible with aging changes (Supplementary Table 1). Although white matter changes were seen in some areas of the 16 control cases, the extent of the comparison of white matter pathology in RDP versus controls could not be done with the same anatomical accuracy.

### Localization of Na,K-ATPase α3 protein

**Fig. 8:**
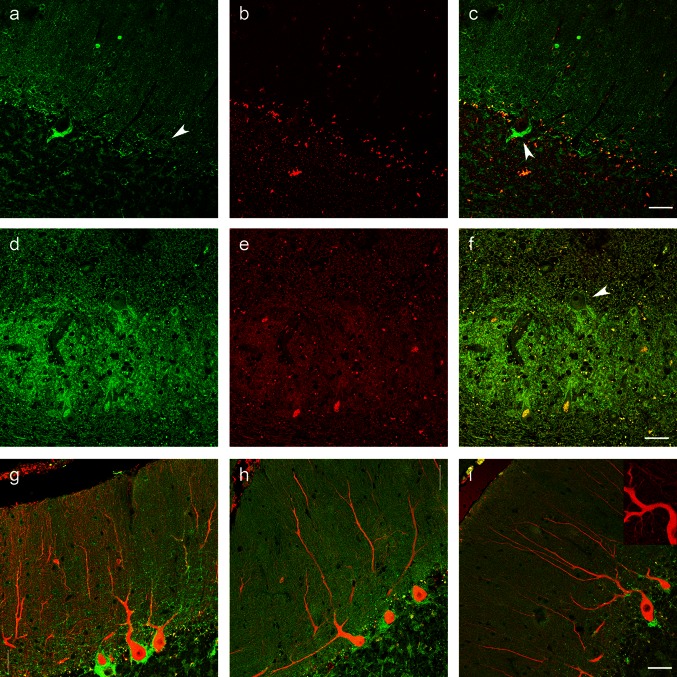
Immunolabeling of Na,K-ATPase α3 subunit, the protein product of ATP1A3. **a**–**c** Control cerebellar cortex showing diffuse fluorescence for α3 (**a**, **c**, *green*) in the molecular layer, with more prominent labeling of molecular layer neurons (*arrowhead*, **a**) and basket cell processes surrounding the Purkinje cell bodies (*arrowhead*, **c**). In the granular layer, fluorescence for α3 (**a**, **c**, *green*) lightly ring-labeled the granule neurons and more heavily labeled the glomeruli. Fluorescence in the red channel (**b**, **c**) was due to lipofuscin. **d**–**f** Diffuse α3 fluorescence was seen in the tissue surrounding the dentate nucleus, with much brighter signal in the dentate nucleus (**d**, **f**), particularly around large-diameter neurons (*arrowhead*, **f**). Lipofuscin fluorescence was also present, particularly in neuronal somas (**e**, **f**). The panels (**g**–**i**) show control brain (**g**), case 3 unaffected gene carrier (**h**), and case 1 affected gene carrier (**i**). Double label was for calbindin (*red*) and Na,K-ATPase α3 (*green*). An *inset* is included in (**i**) that shows the absence of fluorescence corresponding to the bright green fluorescence in the merged image. This indicates that the green fluorescence is not due to lipofuscin. *Magnification bars* are 50 μm except for the *inset* in (**i**) at 32 μm

Immunolabeling of the ATP1A3 gene product in sections of cerebellum from a control case (Fig. 8a–g), the non-symptomatic mutation carrier (Fig. 8h), and a symptomatic mutation carrier (Fig. 8i) showed a pattern of expression very similar to that seen in previous studies of rodents (discussed below). In cerebellar cortex, brightest signal was from the inhibitory processes of basket cells, but many cerebellar cortex neurons were positive. Large neurons of the dentate nucleus were particularly well stained. In Fig. 8g–i, representative images are shown for control human tissue (age 76), for Case 3 (unaffected carrier), and Case 1 (affected carrier) double-labeled for calbindin to highlight the Purkinje neurons in red. Qualitatively, the distribution was similar. Although the signal may appear less bright in the affected carrier, the epitope is sensitive to conditions and there is insufficient information to conclude that there is a difference in the enzyme’s expression or distribution.

### Molecular genetic analysis

**Fig. 9:**
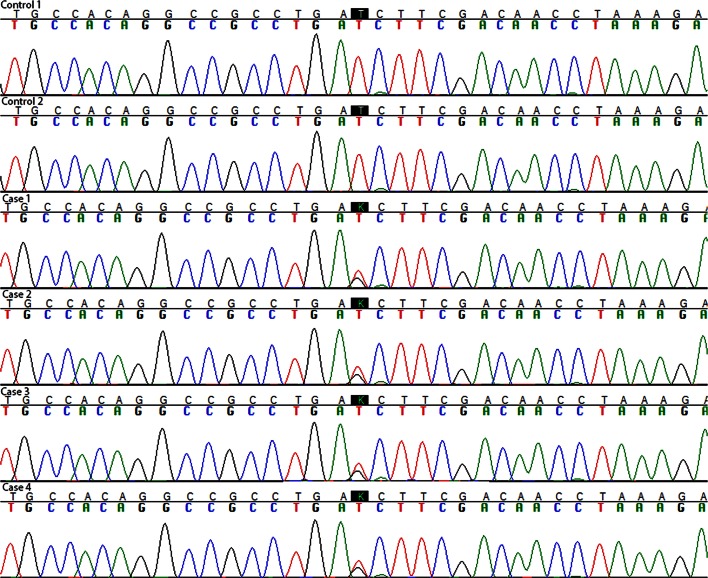
Molecular genetic analysis. An ATC > AGC point mutation resulting in an amino acid substitution of isoleucine to serine (I758S) in the *ATP1A3* gene in the four siblings but not in control cases was observed. The *K in the black box* is a code indicating that both *T* (thymidine, normal allele) and *G* (guanidine, mutant allele) are present as expected for a dominant mutation

Molecular genetic analysis revealed an ATC > AGC point mutation predicting an amino acid substitution of isoleucine to serine (I758S) in the *ATP1A3* gene (Fig. 9). *APOE* results are listed in Table 1. None of the I758S mutation carriers had an *APOE 4* allele, which is a risk factor for AD. See Supplementary Table 1 for control data.

## Discussion

The present study, the first of its kind, focuses on the neuropathology of four siblings, three affected by RDP and one not manifesting clinical signs, all carriers of the I758S mutation. The 4 siblings and 16 controls had the following pathologic changes in common: (1) a mild to moderate degree of cerebrovascular disease and (2) neurodegenerative changes of the Alzheimer type. In the four siblings and controls, the Alzheimer pathology involved predominantly neocortex and hippocampus. All were classified as having mild to moderate Alzheimer disease changes. In addition, in the three symptomatic gene carriers, atrophy and severe neuronal loss were observed in areas free of cerebrovascular and Alzheimer disease pathology. These were globus pallidus, subthalamic nucleus, red nucleus, inferior olivary nucleus, cerebellar Purkinje cell layer, granule cell layer, and dentate nucleus. When mild neuronal loss was observed in any gray matter structure, both in symptomatic gene carriers and controls, it was not interpreted as being significant for RDP. In the cortex and in areas free of Alzheimer pathology, immunohistochemistry for tau, α-synuclein, TDP-43, and ubiquitin failed to show any intracytoplasmic or intranuclear inclusions. In spite of the name of the disorder, “rapid-onset dystonia-parkinsonism,” pathologic changes were not detected in the substantia nigra. In addition, white matter tracts of the cerebrum, including cortico-cortical, cortico-spinal, cortico-pontine, cortico-bulbar, thalamo-cortical, and cortico-subcortical fibers, showed axon and myelin losses. Furthermore, efferent fibers of the dentate nucleus and the intrafoliar white matter bundles of the cerebellum were affected, while in the spinal cord, the ascending sensory fibers of the dorsal columns were reduced. The clinicopathologic correlate of the dorsal column alterations remains unclear at this time.

The availability of the four I758S mutation carriers’ brains has offered the unique opportunity to study, under the same conditions and within a single laboratory, siblings with the same mutation, similar ages at death, and who have undergone extensive longitudinal clinical evaluations. Given the rarity of RDP, it was decided to follow a dissection protocol aimed at obtaining half of the brain for future biochemical analysis. However, in so doing, our ability of comparing the right and left side to establish specific clinicopathological correlations and analyze asymmetry of anatomical lesions was lost. A significant challenge in this study has been the fact that the neuropathologic investigation has taken place many decades after the clinical phenotype has manifested itself. Thus, the neuropathologic analysis had to take into consideration co-morbidities associated with old age, namely vascular pathology and neurodegeneration. The presence of Alzheimer pathology, including neuronal loss, has been an obstacle for investigating possible RDP changes in the cerebral cortex.

In spite of the fact that the four siblings carried the same genetic mutation, clinical signs of RDP developed in only three of them and in those three, we identified anatomical alterations. At this time, it is not clear what the interaction might be between genetic defect and other genetic and/or environmental elements for the development of the full clinical phenotype. In addition, whether the cell losses attributed to the genetic defect are preceding the clinical symptoms or are occurring as a result of the movement disorder remains to be understood. It is of interest that among the three RDP-affected siblings, the twins had a very similar clinical and pathologic phenotype including age at onset, while the third subject’s clinical phenotype presented more than a decade later than the twins. In addition, the presence of AD changes in all four gene carriers raises the question as to whether the mutation in the *ATP1A3* gene represents a risk factor for the development of Alzheimer disease.

To determine which anatomical structures are affected in the earliest stages of RDP, it would be important to obtain imaging in gene carriers before the onset of symptoms and in the early stages of RDP. Data along these lines are limited; however, recent imaging results by Whitlow and colleagues in RDP-affected male patients aged 26, 56, and 63 are consistent with some of the findings of the present study [37]. In fact, there were statistically significant gray matter volume decreases in RDP patients compared to controls in the dentate nucleus. Fractional anisotropy was reduced among RDP patients compared to controls in the superior cerebellar peduncle, dentate nucleus, parahippocampal gyrus, thalamus, basal ganglia (caudate nucleus, putamen, and globus pallidus), internal and external capsules, and the corpus callosum. Findings of a reduction in fractional anisotropy in the basal ganglia and the white matter pathways that carry information between brain areas supports the neuropathologic findings presented here and supports further the proposed abnormal circuitry in the basal ganglia and cerebellum.

Detailed neuropathologic reports of other primary dystonias are limited. However, nuclei implicated in RDP have also been implicated in other dystonias. In pallidoluysian atrophy, severe atrophy of the subthalamic nucleus and globus pallidus was reported [38]. A diffusion tensor imaging (DTI) and neuropathological study in spasmodic dysphonia found decreased fractional anisotropy in the internal capsule and cortico-bulbar/cortico-spinal tracts, and water diffusivity was increased in the lentiform nucleus, thalamus, and cerebellum [34]. Neuropathologic examination revealed a loss of axonal density and myelin content in the internal capsule. In Meige syndrome, neuronal losses occurred in the substantia nigra, locus coeruleus, midbrain tectum, and dentate nucleus of the cerebellum [20]. Although there are phenotypic and genetic variations among the many dystonias, several key nuclei and white matter tracts appear to be affected.

Taking these studies together, data from imaging and neuropathology seem to point to the involvement of specific anatomical gray matter areas and white matter pathways. In this group of movement disorders, genetic and environmental factors may play a role in causing the clinical and anatomical heterogeneity. The present data underscore the necessity of more systematic quantitative studies of neuron number in areas that neuropathologic and neuroimaging studies seem to indicate to be most relevant in disease pathogenesis of dystonia. Our current analysis has yielded some important evidence to direct future quantitative studies aimed at assessing neuron number and dendritic morphology in brain areas identified as vulnerable in RDP. The current limitation of the present study is the lack of power resulting from the number of available cases.

Currently available data in rodents support our findings in view of the fact that high α3 immunostaining has been found in striatum, globus pallidus, subthalamic nucleus, substantia nigra, cerebellum, red nucleus, and the pontine nuclei [4, 27, 29]. In situ hybridization data for the mouse brain in the Allen Brain Atlas (http://www.brain-map.org) also show intense ATP1A3 mRNA reactivity in cell somas in cerebellum, dentate nucleus, red nucleus, inferior olivary nucleus, and other regions of interest, supporting the possibility that pathology is intrinsic to cells that express the mutated enzyme. Different classes of neurons can differ in the Na,K-ATPase isoforms they express, the expression levels, and their physiological impact [1, 29], but it is not given that a high level of expression of ATP1A3 is the most important factor. The presence of pathology may relate more to the interaction of reduced Na,K-ATPase activity with the physiology of a particular cell. It is even possible that cells with pronounced pathology were injured by excessive excitatory input or a deficit of inhibitory input resulting from mutated Na,K-ATPase in otherwise-healthy presynaptic cells. The important findings are the correlation of symptoms of RDP with demonstrable pathology and the scarcity of pathology in the patient with no symptoms, and that the identified neuronal populations are part of complex motor and sensory loops. Our data on the localization of the Na,K-ATPase α3 protein in affected and control subjects strengthen our results related to the involvement of the cerebellum in RDP. Additional studies will be carried out in areas that we have identified as potentially vulnerable in RDP.

In summary, with the present study of four *ATP1A3* gene carriers, we have identified RDP-associated pathology in neuronal populations in the globus pallidus, subthalamic nucleus, red nucleus, inferior olivary nucleus, cerebellar Purkinje cell layer, granule cell layer, and dentate nucleus. Their involvement would cause an interruption of cerebral and cerebellar connections that are essential for the maintenance of motor control. RDP may be due to dysfunctional regulatory loops such as cortico-striato-pallido-thalamo-cortical and cerebello-thalamo-cortical pathways, and additional brainstem dentatorubral-pallidal and dentato-olivary pathways, with abnormalities in multiple relay stations.

## Electronic supplementary material

Table 1.Demographic information and neuropathologic analysis for control cases.

Table 2.Psychiatric Status of each subject.

Table 3.Cognitive Status of cases 3 and 4.

Below is the link to the electronic supplementary material.

Supplementary material 1 (PDF 82 kb)

Supplementary material 2 (PDF 56 kb)

Supplementary material 3 (PDF 74 kb)
