# Supplementary material for: Rapid-onset dystonia-parkinsonism associated with the I758S mutation of the ATP1A3 gene: a neuropathologic and neuroanatomical study of four siblings
Source: Acta Neuropathol. 2014 May 7;128(1):81–98. doi: 10.1007/s00401-014-1279-x (PMC4059967; doi:10.1007/s00401-014-1279-x)
Supplement: Supplementary file 1 — Supplementary material 1 (PDF 82 kb) [file 401_2014_1279_MOESM1_ESM.pdf]

Supplementary Table 1. Neuropathologic and Alzheimer changes in controls.

	Control 1-1	Control 1-2	Control 1-3	Control 2-1	Conrol 2-2	Control 2-3	Conrol 2-4	Control 2-5	Control 3-1	Control 3-2	Control 3-3	Control 3-4	Control 4-1	Control 4-2	Control 4-3	Control 4-4
**Gender**	F	M	F	F	F	F	M	F	M	F	F	F	F	F	M	F
**Age at death**	88	89	83	99	79	94	84	81	87	74	83	80	88	81	80	80
**PMI (hours)**	11.7	15.1	6.1	10.4	3	20	3.2	17.7	4.7	5.5	4.3	16.9	48	unknown	unknown	13.5
**Brain weight (grams)**	920	1,205	1,310	1,130	1,174	990	1,283	1,130	1,230	990	1,124	1,084	1,300	1,150	1,268	1,141
**APOE**	unavailable	34	34	34	33	34	34	33	33	unavailable	34	33	24	24	33	34
**Alzheimer disease changes (A, B, C)**	3,2,1	3,2,1	3,2,1	3,2,2	3,2,2	3,2,2	3,2,2	3,2,2	3,2,3	3,2,3	3,2,3	unclassifiable	3,2,2	3,2,2	3,2,2	3,2,2
**Superior frontal gyrus & anterior cingulate gyrus**	1	2	1	2	1	2	2	1	2	2	2	1	2	2	1	1
**Caudate nucleus & putamen**	1	1	1	2	1	2	1	0	1	1	1	2	2	2	0	1
**Globus pallidus**	1	1	1	1	1	2	2		1	1	1	1	2	2	1	1
**Substantia innominata**	1	1	1	1	2	2	2	1	3	1	2	2	3	2	0	1
**Amygdala**	3	2	1	2	1	3	1	1	3	2	2	2	2	3	0	2
**Superior and middle temporal gyrus**	1	2	1	1	1	2	2	2	2	2	2	2	2	2	0	2
**Subthalamic nucleus**	1	2	2	2	1	1	1	0	1	1	1	0	1	2	0	2
**Thalamus**	1	2	2	2	1	1	1	0	1	1	1	0	2	2	0	2
**Hippocampus, CA1 - CA4**	2	2			1	1	2	2	1			1	2	2	0	2
**Hippocampus, Molecular layer**	0	2	1	1	0	0	0	0	0	0	0	0	0	0	0	0
**Hippocampus, Dentate Gyrus**	0	1	0	0	1	2	1	0	0	0	0	1	1	1	0	2
**Subiculum**	3	3	0	1	1	3	2	1	3	2	2	1	2	2	0	2
**Entorhinal Cortex**	3	2	1	2	1	3	2	1	3	2	2	2	2	2	0	3
**Superior parietal lobule**	2	2	1	2	1	2	3	1	2	2	2	2	2	2	0	1
**Calcarine cortex**	1	2	1	2	1	1	2	1	2	2	2	1	1	1	0	1
**Cerebellum, molecular layer**	1	2	1	1	1	2	0	0	1	0	0	2	0	2	0	1
**Cerebellum, Purkinje cell layer**	0	2	1	2	1	3	2	1	2	1	2	2	2	2	0	2
**Cerebellum, granule cell layer**	0	2	1	2	1	2	0	0	1	0	0	2	0	2	0	2
**Cerebellum, dentate nucleus**	2	0	1	2	1	1	1	1	0	1	2	1	1	2	0	2
**Cerebellum, white matter**	0	2	0	2	0	0	0	0	0	0	0	0	0	0	0	0
**Midbrain, superior colliculus**	0	2	0	0	0	0	0	0	0	0	1	0	0	0	0	0
**Midbrain, periaqueductal grey**	0	3	0	0	0	0	0	0	0	0	0	0	0	0	0	0
**Midbrain, substantia nigra**	2	1	0	0	1	1	2	2	1	2	2	0	1	1	0	1
**Midbrain, red nucleus**	0	0	1	1	0	0	0	0	0	0	0	0	0	0	0	0
**Pons, locus coeruleus**	1	1	0	0	1	2	1	1	0	2	1	2	2	3	0	3
**Pons, pontine nuclei**	0	0	2	3	0	0	1	0	0	0	0	0	0	0	0	0
**Pons, reticular formation**	0	0	0	0	0	0	0	0	0	0	0	0	0	0	0	0
**Medulla, dorsal motor nucleus of the vagus nerve**	0	0	0	0	0	1	1	0	0	0	2	2	2	3	0	2
**Medulla, hypoglossal nucleus**	0	0	1	2	0	1	1	0	0	0	0	2	2	3	0	2
**Medulla, inferior olivary nucleus**	1	0	0	2	1	1	2	2	0	1	2	2	1	2	0	2
**Middle frontal gyrus**	1	2	1	2	1	2	2	1	2	n/a	n/a	1	2	2	0	1
			1	1												

Areas of neuronal loss and gliosis AND no associated AD related pathology (neuritic plaques, diffuse plaques, neurofibrillary tangles, neuropil threads, tau- ir neurons)
Plaques (Neuritic and Diffuse)
Plaques (Neuritic and Diffuse) & Tangles
Tangles, only

Degree of neuron loss and gliosis in brain regions: none = 0, mild = 1, moderate = 2, severe = 3

Article title: Rapid-onset dystonia-parkinsonism associated with the I758S mutation of the ATP1A3 gene: A neuropathologic and neuroanatomical study of four siblings

Journal name: Acta Neuropathologica

Authors: Adrian L. Oblak, Ph.D., Matthew C. Hagen, M.D., Ph.D., Kathleen J Sweadner, Ph.D.,
Ihtsham Haq, M.D., Christopher T. Whitlow, M.D., Ph.D., Joseph A. Maldjian, M.D., Francine Epperson, Jared F. Cook, M.A., Mark Stacy, M.D., Jill R. Murrell, Ph.D., Laurie J Ozelius, Ph.D., Allison Brashear, M.D., Bernardino Ghetti, M.D.

Corresponding author:
Bernardino Ghetti, MD
Indiana University School of Medicine
Department of Pathology and Laboratory Medicine
bghetti@iupui.edu
