# Supplementary material for: Rapid-onset dystonia-parkinsonism associated with the I758S mutation of the ATP1A3 gene: a neuropathologic and neuroanatomical study of four siblings
Source: Acta Neuropathol. 2014 May 7;128(1):81–98. doi: 10.1007/s00401-014-1279-x (PMC4059967; doi:10.1007/s00401-014-1279-x)
Supplement: Supplementary file 2 — Supplementary material 2 (PDF 56 kb) [file 401_2014_1279_MOESM2_ESM.pdf]

Supplementary Table 2. Psychiatric Status of each subject.

Case	Age at visit	Gender	Age at onset of motor symptoms	Self-Report	DSM IV diagnosis	Specific psychotic symptoms
1	80	F	28	Depression, Anxiety	N/A	N/A
2	81	F	25	Depression, Anxiety	N/A	N/A
3	84	F	N/A	Depression	None	N/A
4	83	M	45	Depression, Anxiety	Psychotic disorder NOS; panic disorder without agoraphobia; alcohol abuse	Paranoid delusions, thought insertion, thought broadcasting, thought withdrawal

N/A = Not Applicable, NOS = Not otherwise specified

<u>Article title</u>: Rapid-onset dystonia-parkinsonism associated with the I758S mutation of the ATP1A3 gene: A neuropathologic and neuroanatomical study of four siblings

Journal name: Acta Neuropathologica

<u>Authors</u>: Adrian L. Oblak, Ph.D., Matthew C. Hagen, M.D., Ph.D., Kathleen J Sweadner, Ph.D.,

Ihtsham Haq, M.D., Christopher T. Whitlow, M.D., Ph.D., Joseph A. Maldjian, M.D., Francine Epperson, Jared F. Cook, M.A., Mark Stacy, M.D., Jill R. Murrell, Ph.D., Laurie J Ozelius, Ph.D., Allison Brashear, M.D., Bernardino Ghetti, M.D.

Corresponding author:
Bernardino Ghetti, MD
Indiana University School of Medicine
Department of Pathology and Laboratory Medicine
bghetti@iupui.edu
