# Supplementary material for: Rapid-onset dystonia-parkinsonism associated with the I758S mutation of the ATP1A3 gene: a neuropathologic and neuroanatomical study of four siblings
Source: Acta Neuropathol. 2014 May 7;128(1):81–98. doi: 10.1007/s00401-014-1279-x (PMC4059967; doi:10.1007/s00401-014-1279-x)
Supplement: Supplementary file 3 — Supplementary material 3 (PDF 74 kb) [file 401_2014_1279_MOESM3_ESM.pdf]

Supplementary Table 3. Cognitive Status

Test	Case 3	Case 4
**IQ Estimate (1)**	SS / Pr	SS / Pr
Raven's Progressive Matrices	75 / 5th (Borderline)	78 / 7th (Borderline)
**Memory and Learning (2)**	ScS / Pr	ScS / Pr
WRAML2 Picture Memory	--	8 / 25th (Average)
WRAML2 Picture Memory Delayed Recognition	--	5 / 5th (Borderline)
WRAML2 Design Memory	6 / 9th (Borderline)	5 / 5th (Borderline)
WRAML2 Design Memory Delayed Recognition	6 / 9th (Borderline)	9 / 37th (Average)
WRAML2 Verbal Learning	9 / 37th (Average)	8 / 25th (Low Average)
WRAML2 Verbal Learning Delayed Recall	7 / 16th (Low Average)	8 / 25th (Low Average)
WRAML2 Verbal Learning Delayed Recognition	10 / 50th (Average)	6 / 9th (Below Average)
**Psychomotor Speed and Attention**	T or Z score / Pr	T or Z score / Pr
Trail Making Test Part A (3) *	31 / 3rd (Mild-Moderate Impairment)	40 / 16th (Below Average)
SDMT-Written (4)	--	-3.00 / 1st (Very Low)

SDMT-Oral (4)	--	-2.70 / 1st<br>(Very Low)
**Executive Functioning**	T score / Pr	T score / Pr
Trail Making Test Part B (3) *	--	35 / 7th<br>(Mild Impairment)
COWA Semantic Verbal Fluency (5) *	35 / 7th<br>(Mild Impairment)	28 / 2nd<br>(Moderate Impairment)
COWA Linguistic Verbal Fluency (5) *	37 / 9th<br>(Mild Impairment)	28 / 2nd<br>(Moderate Impairment)

Pr = Percentile rank

SS = Standard Score (mean = 100, standard deviation = 15)

ScS = Scaled Score (mean = 10, standard deviation = 3)

T score (mean = 50, standard deviation = 10)

Z score (mean = 0, standard deviation = 1)

WRAML2 = Wide Range Assessment of Memory and Learning, Second Edition

SDMT = Symbol Digit Modalities Test

COWA = Controlled Oral Word Association

* T scores derived from demographically adjusted neuropsychological normative data (6)

Article title: Rapid-onset dystonia-parkinsonism associated with the I758S mutation of the ATP1A3 gene: A neuropathologic and neuroanatomical study of four siblings
Journal name: Acta Neuropathologica

Authors: Adrian L. Oblak, Ph.D., Matthew C. Hagen, M.D., Ph.D., Kathleen J Sweadner, Ph.D., Ihtsham Haq, M.D., Christopher T. Whitlow, M.D., Ph.D., Joseph A. Maldjian, M.D., Francine Epperson, Jared F. Cook, M.A., Mark Stacy, M.D., Jill R. Murrell, Ph.D., Laurie J Ozelius, Ph.D., Allison Brashear, M.D., Bernardino Ghetti, M.D.

Corresponding author:
Bernardino Ghetti, MD
Indiana University School of Medicine
Department of Pathology and Laboratory Medicine
bghetti@iupui.edu

Reference List

(1) Raven JC. Raven's Progressive Matrices and Coloured Progressive Matrices with Research Supplement. (2000) Harcourt Assessment, San Antonio, USA

(2) Sheslow D, Adams W. Wide Range Assessment of Memory and Learning, Second Edition. ed. (2007) Wide Range Inc., Florida, USA

(3) Washington DC- Adjutant General's Office WD. Army Individual Test Battery. Manual of directions and scoring. (1944)

(4) Smith A. Symbol Digit Modalities Test: Manual. (2007) Western Psychological Services, Los Angeles, USA

(5) Spreen O, Strauss E. A Compendium of Neuropsychological Tests. (1998) Oxford University Press, New York, USA

(6) Heaton RK, Miller W, Taylor MJ, Grant I. (2004) Revised Comprehensive Norms for an Expanded Halstead-Reitan Battery: Demographically Adjusted Neuropsychological Norms for African American and Caucasian Adults. Psychological Assessment Resources, Inc. USA
